# Fibromyalgia in patients infected with HTLV-1 and HTLV-2

**DOI:** 10.3389/fmed.2024.1419801

**Published:** 2024-08-23

**Authors:** Bianca Lumi Inomata Silva, Francisco Erivan da Cunha Rodrigues, Márcio Yutaka Tsukimata, Bruno José Sarmento Botelho, Luciana Cristina Coelho Santos, Gabriel dos Santos Pereira Neto, Aline Cecy Rocha Lima, Natália Pinheiro André, Sarah Marques Galdino, Danniele Chagas Monteiro, Gilberto Toshimitsu Yoshikawa, Leonardo Teixeira Mendonça, Juliana Lasmar Ayres do Amaral, Rosana de Britto Pereira Cruz, Débora Oliveira Onuma, Antonio Carlos Rosário Vallinoto, Bárbara Nascimento de Carvalho Klemz, Izaura Maria Vieira Cayres Vallinoto

**Affiliations:** ^1^Laboratório de Virologia, Instituto de Ciências Biológicas, Universidade Federal do Pará, Belém, Brazil; ^2^Faculdade de Medicina, Instituto de Ciências Médica, Universidade Federal do Pará, Belém, Brazil; ^3^Programa de Pós-graduação em Biologia de Agentes Infecciosos e Parasitário, Universidade Federal do Pará, Belém, Brazil

**Keywords:** fibromyalgia, pain, virus, infection, HTLV-1, HTLV-2

## Abstract

**Background:**

Reports on the association between HTLV-2 infection and the development of diseases in the human host are rare, which has led the scientific community to believe that HTLV-2 is not an important etiological agent of lymphoproliferative or neurodegenerative disorders, which is the case for HTLV-1. In the present study, we demonstrated cases of fibromyalgia in HTLV-1 carriers and, in an unprecedented finding, in two patients with confirmed HTLV-2 infection.

**Methods:**

A total of 957 individuals visited the Virology Laboratory at the Federal University of Pará for screening and confirmation tests for HTLV-1/2 infection. Individuals with confirmed HTLV-1 and HTLV-2 infection were clinically evaluated for signs and symptoms associated with infection.

**Results:**

Sixty-nine individuals (7.2%) were identified as positive for HTLV infection, with 56 confirmed cases of HTLV-1 infection (5.9%), 12 cases of HTLV-2 infection (1.2%) and one case classified as undetermined (0.1%). Sixteen (23.2%) of these patients presented with rheumatological signs and complained of diffuse pain throughout the body; 12 of whom were infected by HTLV-1 (75%) and 4 were infected by HTLV-2 (25%). After anamnesis and careful evaluation, four patients were diagnosed with fibromyalgia, two of whom were infected by HTLV-1 (16.7%; 2/12) and two by HTLV-2 (50%; 2/4). The clinical follow-up and laboratory analysis results are reported in detail in this paper.

**Conclusion:**

Considering the clinical cases presented herein as the first reports of patients with HTLV-2 infection with clinical symptoms of fibromyalgia, the importance of further studies on the pathogenicity of HTLV-2, similar to what have already been performed for HTLV-1, is highlighted. Our results also confirm previous evidence of an association between HTLV-1 infection and fibromyalgia.

## Introduction

Human T-lymphotropic viruses 1 and 2 (HTLV-1 and HTLV-2, respectively) are retroviruses belonging to the genus *Deltaretrovirus*, family *Retroviridae* (1). HTLV-1 was isolated for the first time in 1980 in the USA from a lymphoblastoid cell line obtained from a patient with cutaneous T-cell lymphoma (2). HTLV-2 was isolated in 1982 from a patient with hairy cell leukemia (3).

Although HTLV-1 is widely associated with lymphoproliferative, neurodegenerative and inflammatory diseases (4), HTLV-2 has been associated with rare cases of neurological disease similar to HTLV-1-associated myelopathy (HAM) (5, 6) and the presence of atypical lymphocytes (7).

Fibromyalgia is a chronic and diffuse musculoskeletal pain syndrome that was first described in the 19th century (8). Initially, fibromyalgia was called “pain syndrome” by Graham (9), as patients with pain were observed without an organic disease that could justify this symptom. The term “fibromyalgia” was implemented only when “pain points” were discovered by Smythe and Moldofsky (10). In 1990, the American College of Rheumatology (ACR) began to develop diagnostic criteria for this disease based on anamnesis and physical examination findings since laboratory and imaging tests do not reveal pathognomonic abnormalities. These criteria have periodically been updated, but investigations have always prioritized clinical examination findings.

The cause of fibromyalgia is still unknown, but there are hypotheses that a viral infection may be responsible for its onset (6, 11). Oltra et al. (12), when seeking to associate fibromyalgia with the presence of retrovirus infection, did not observe evidence that the disease was associated with HTLV infection. However, fibromyalgia was subsequently observed at a high prevalence in people living with HTLV-1, but not in those living with HTLV-2 (13). In a recent meta-analysis study Schierhout et al. (4) showed that HTLV-1 was associated with several other potentially inflammatory conditions, amongst them the fibromyalgia.

In the present study, we report the cases of four patients with a clinical diagnosis of fibromyalgia according to ACR criteria who, after laboratory tests, were confirmed to be infected by HTLV-1 and HTLV-2 and experienced progression of their clinical condition over time. To our knowledge, this is the first report in the literature of fibromyalgia in people living with HTLV-2.

## Methods

### Study design and sampling

During the period from April 2022 to December 2023, a total of 957 individuals visited the Virology Laboratory at the Federal University of Pará in search of screening and confirmation tests for HTLV-1/2 infection. This group was composed by people, residing in the metropolitan area of the Belém capital, who voluntarily sought a screening and confirmatory diagnosis of the infection. Individuals with confirmed HTLV-1 and HTLV-2 infection were subsequently summoned for an initial consultation with a nurse, where they were then examined and given guidance about the infection. These individuals were then invited to join the Care Service for People Living with HTLV (SAPEVH) to receive support and monitoring from a multidisciplinary team.

### Ethical considerations

This study was reviewed and approved by the Human Research Ethics Committee of the Health Sciences Institute of the Federal University of Pará (CAAE: 27290619.2.0000.0018 and CAAE: 71261523.1.0000.0018) in accordance with the directives of the Declaration of Helsinki. The objectives of the project were explained to all participants at the time of inviting to join the Care Service for People Living with HTLV (SAPEVH). After the explanation, an informed consent for the publication of these case reports was obtained from the patients.

### HTLV serological diagnosis

Plasma detection of total anti-HTLV-1/2 antibodies was performed using an ELISA (Murex HTLV-I + II, DiaSorin, Dartford, United Kingdom). Samples with reactive or indeterminate results (cutoff = 0.284) were subjected to confirmatory Western blotting (HTLV Blot 2.4, MP Diagnostics, Singapore, Republic of Singapore), following the manufacturer’s protocol.

### HTLV molecular diagnosis

Serum positive samples were subsequently subjected to qPCR using the TaqMan system (Applied Biosystems, Foster City, CA) on the Applied Biosystems StepOne Plus Real Time PCR platform, as described below (14).

Human albumin gene was used as an endogenous reaction control, and the viral gene pol (186 bp) of HTLV-1 and tax (75 bp) of HTLV-2 were used as viral confirmation and molecular differentiation targets (15). Each reaction contained 12.5 μL of TaqMan Universal PCR Master Mix (2X) (Applied Biosystems, Foster City, United States), 6.0 μL of ultrapure water, 0.5 μL of each primer, 0.5 μL of each probe and 5.0 μL of DNA, resulting in a total volume of 25 μL. The following temperature cycles were used: 95°C for 10 min, followed by 45 cycles of 95°C for 15 s and 60°C for 1 min for primer and probe binding.

The primers were used in the reactions were: 5′-CCCTACAATCCAACCAGCTCAG-3′ (HTLV-1F), 5′-GTGGT GAAGCTGCCATCGGGTTTT-3′ (HTLV-1R), 5′-CGATTGTGTAC AGGCCGATTG-3′ (HTLV-2F), 5′-CAGGAGGGCATGTCGAT GTAG-3′ (HTLV-2R), 5′-GCTGTCATCTCTTGTGGGCTGT-3′ (Albumin F), and 5′-AAACTCATGGGAGCTGCTGGTT-3′ (Albumin R). The probe sequences were: FAM-5′- CTTTACTGACAAACCCGACCTACCCATGGA-3′-MGB (HTLV-1), FAM-5′-TGTCCCGTCTCAGGTGGTCTATGTTCCA-3′-MGB (HLTV-2) and FAM-5′- CCTGTCATGCCCACACAAATCTC-3′-MGB (Albumin).

## Results

Of the 957 individuals tested, 69 (7.2%) were positive for HTLV-1/2 infection, with 56 confirmed cases of HTLV-1 infection (5.9%), 12 cases of HTLV-2 infection (1.2%) and 1 case classified as indeterminate (0.1%).

From the initial assessment of the 69 people living with HTLV (PVHTLV) conducted by the nurse at the initial consultation, it was observed that 16 (23.2%) patients had rheumatological signs and complaints of diffuse pain throughout the body, 12 of whom had HTLV-1 infection (75%) and 4 of whom had HTLV-2 infection (25%). These individuals were then referred for follow-up with the team’s rheumatologist. No symptoms were reported in the other 53 individuals. After anamnesis and careful evaluation following the American College of Rheumatology (ACR) criteria, four patients were diagnosed with fibromyalgia, two infected by HTLV-1 (16.7%; 2/12) and two infected by HTLV-2 (50%; 2/4), as described below.

### Patient #1

A 56-year-old female with a confirmed diagnosis of HTLV-1 infection in 2022 was diagnosed with fibromyalgia. In April 2022, she attended her first consultation with a rheumatologist, presenting diffuse pain throughout his body, which had been present for approximately 10 years. Pain was located in the wrists, fingers, shoulders, ankles, and lumbar region (irradiating to the right lower limb), with greater intensity at the end of the day (visual numeric pain scale (VNS) = 7). She reported difficulty getting out of bed and getting up from a chair (she needed to support herself to do so). She had morning stiffness >30 min (mostly because of pain). She reported the use of cyclobenzaprine and gabapentin, in addition to warm water compresses, to improve her condition. The pain worsened during cold periods and upon exertion. She complained of nonrestorative sleep.

The patient underwent physiotherapy and hydrotherapy, which reduced pain in her joints, but she was unable to continue the therapy. Electroneuromyography of the lower limbs (ENMG MMII) carried out in 2021 revealed myelopathy from L2 to S1 of probable inflammatory etiology in the chronic phase. When asked, she denied cases of HTLV infection in her family, as well as the use of legal or illicit drugs, but she mentioned being sedentary. She was hypertensive, for which she was taking losartan 50 mg/day, and she had recurrent urinary tract infections (she was being monitored by an infectious disease specialist). She was taking 300 mg/day gabapentin, which was prescribed by the clinician due to her spinal condition.

On physical examination, there was pain on palpation of the hand joints but no synovitis. A squeeze test was positive. There was also pain on palpation of the paravertebral lumbar region, with an accentuation of lumbar lordosis that worsened with hyperextension. Crepitus was noted in both knees, while arthritis was absent. There was pain on palpation of the metatarsophalangeal (MTP) joints. A squeeze test was positive bilaterally. Lower limb edema (+/4+) was noted. There was pain on shoulder mobilization, but range of motion (ROM) was preserved. Given these findings, the diagnostic hypotheses were joint pain with etiology to be clarified and fibromyalgia. Laboratory tests (Table 1) were requested [blood count, erythrocyte sedimentation rate (ESR), C-reactive protein (CRP), rheumatoid factor (RF), antinuclear factor (ANA), total cholesterol, low- and high-density lipoproteins (LDL and HDL, respectively), triglycerides, and glucose], as were X-ray of the hands, feet and spine.

**Table 1:** The results of exams carried out on patients diagnosed with fibromyalgia.

Patients	Exams			
Patient #1	Laboratory test results (04-25-2022): LDL = 108.8 mg/dL; triglycerides = 128 mg/L; glucose = 95 mg/dL; rheumatoid factor < 20 UI/mL; C-reative protein <8 mg/dL; antinuclear factor = 1/80; ESR = 63 mm/h; total cholesterol = 169 mg/dL; HDL = 36 mg/dL hemoglobin = 14.7 g/dL; hematocrit = 45.1%; leukocytes = 6,590 mm^3^; neutrophils = 54%; lymphocytes = 37.7 mm^3^ platelets = 186.000.mm^3^ Lumbar spine X-ray (05–112,022): reduction in L5-S1 disc space. Feet X-ray (05-25-2022): plantar enthesophytes and Achilles tendonitis. Hand X-ray (05-06-2022): no pathological changes. Colonoscopy (10/01/2022): internal hemorrhoids. USG abdomen (12/20/2021): signs of cholecystectomy	Laboratory test results Anti-SSA = negative; anti-SSB = negative; hemoglobin = 13.8 g/dL; hematocrit = 42.8%; leukocytes = 6,470 mm^3^; platelets = 224,000 mm^3^; reticulocytes = 1.7% (absolute value = 79.220); creatinine = 0.76 mg/dL; AST = 22.3 u/L; ALT = 38.1 U/L; ureia = 17 mg/dL; Routine urine test (EAS): leukocytes = 2/field; red cells 1/field; urine culture <1,000 colony forming units - CFU/mL.	Laboratory test results ESR = 20 mm/h; C-reactive protein = 12 mg/dL; rheumatoid factor = 8 UI/mL; glycated hemoglobin = 5% HDL = 59 mg/dL; total cholesterol = 227 mg/dL; LDL = 138 mg/L and triglycerides = 116 mg/dL	Laboratory test results Hemoglobin = 13.9 g/dL; hematocrit = 43%; leukocytes = 8,400 mm^3^; platelets = 171,000 mm^3^; ureia = 24 mg/dL; creatinine = 0,75 mg/dL; AST = 18 U/L; ALT = 23 U/L; alkaline phosphatase = 101.2 U/L; gamma-glutamyl transferase = 16.8 UI/L; DHL = 188 UI/L; Na = 143 mmol/L; K = 4.0 mmol/L; creatine phosphokinase (CPK) = 143 U/L; TSH = 1.96 UI/mL; free T4 = 0.85 ng/dL; Toxoplasmosis IgM = Nonreactive; Toxoplasmosis IgG = 175 UI/mL; VDRL = Nonreactive; HIV-1 e HIV-2 = Nonreactive; anti-HCV = Nonreactive; vitamin B12 = 716 pg./mL (210–980); Parasitológico fezes (PPF) = negative; Routine urine test (EAS): Leucocytes = 0–2/field. Absence of red blood cells. CaT = 9.2; folic acid = 5.64 ng/L; anti-CCP = 1.5 U/mL.
Patient #2	Not performed	Not performed	Not performed	Not performed
Patient #3	Hemoglobin = 13.1 g/dL; hematocrit = 41%; leukocytes = 5,770 mm^3^; platelets = 222,000 mm^3^; ESR = 5 mm/h; C-reative protein <4.0 mg/dL; rheumatoid factor < 3.5 UI/mL and antinuclear factor (ANA) = negative.	Not performed	Not performed	Not performed
Patient #4	(07/13/2022): hemoglobin = 12.2 g/dL; hematocrit = 38.3%; leukocytes = 6,300 mm^3^; platelets = 222,000 mm^3^ glycated hemoglobin = 11.9 mg/dL; glucose = 301 mg/dL; total cholesterol = 286 mg/dL; LDL = 199 mg/dL; urea = 24 mg/dL; triglycerides = 146 mg/dL; creatinine = 0.6 mg/dL; AST = 20 U/L; ALT = 15 U/L; routine urine test (EAS) - glucose +++ and leukocytes 2–4/field.	(07/22/2022): glycated hemoglobin = 8.5 g/dL; ESR = 31 mm/h; glucose = 192 g/dL; total cholesterol = 263 mg/dL; HDL = 57 mg/dL; LDL = 174 mg/dL; triglycerides = 158 mg/dL; Ca total = 10 mg/dL; Mg = 1,8 mmol/L; Na = 136 mEq/L; *p* = 4,0 mg/dL; K = 3,8 mEq/L; C-reactive protein 6.0 mg/dL; rheumatoid factor = 8 UI/mL; routine urine test (EAS) normal and antinuclear factor (ANA) = negative.	(26/10/2022): hemoglobin = 12.4 g/dL; hematocrit = 35.5%; leukocytes 4.700 mm^3^; platelets = 232,000 mm^3^; glycated hemoglobin 7.2 g/dL; glucose 142 g/dL; urea 6 mg/dL; total cholesterol 224 mg/dL; HDL 55 mg/dL; LDL 145 mg/dL; triglycerides = 119 mg/dL and routine urine test (EAS) normal.	(09/15/2023): hemoglobin = 12 g/dL; hematocrit = 39.5%; leukocytes = 4,760 mm^3^; platelets = 182,000 mm^3^; glycated hemoglobin = 11 g/dL; blood glucose = 198.8 g/dL; total cholesterol = 251 mg/dL; triglycerides = 135 mg/dL; urea = 31,8 mg/dL; creatinine = 0.85 mg/dL; ALT = 16.5 UI/L; AST = 16 UI/L; ESR = 19 mm/h; C-reactive protein = 6.0 mg/dL; free T4 = 0,89; rheumatoid factor = 8 UI/mL; TSH = 1.59 mUI/mL and antinuclear factor (ANA) = negative. Bone densitometry (09/18/2023) BMD T score Z score: L1 – L4 (L3) 1,099 0,6 1,5; Colo 0,883 0,3 1,2; Total 1,051 0,9 1,5

In June 2022, the patient returned with exam results, and her joint pain had partially improved at that time. The complaint of low back pain remained unchanged, with worsening with exertion (VNS = 8). She reported morning stiffness (30 to 60 min). Her condition improved with rest and sporadic use of orphenadrine citrate. On physical examination, she presented pain upon mobilization of the lumbar spine, especially during extension, as well as pain on palpation of the cervical, dorsal and lumbar paravertebral regions. The Lasègue test was negative. The diagnostic hypotheses were plantar fasciitis, osteoarthritis, low back pain, and mechanical neck pain.

The therapeutic approach was referral to motor physiotherapy and integrative therapy and guidance on the use of appropriate footwear. Autoantibody testing was requested due to antinuclear antibody (ANA) positivity.

In October 2022, the patient returned with the requested test results. She reported pain in her lower back, hips and lower limbs, which worsened after stopping the use of medications (VNS = 9–10). She also complained of pain and paresthesia in her hands and had experienced difficulty carrying objects for a month. Additionally, she had experienced pain in her wrists and knees. She denied morning stiffness. She reported that she stopped the medication cyclobenzaprine 10 mg/day because the prescription expired and gabapentin 300 mg/day because of financial reasons.

On physical examination, she presented pain on palpation of the joints of her hands. Synovitis in the left wrist was noted. A squeeze test was positive. Tinel tests were positive on the right, and Phalen tests were positive bilaterally. She reported pain on palpation of the paravertebral lumbar region, with accentuation of lumbar lordosis. She presented worsening hyperextension and pain on palpation of the metatarsophalangeal joints. A squeeze test of the feet was positive bilaterally. Lower limb edema (+/4+) was noted. She also presented pain on palpation of the medial and proximal surface of the right tibia in the area of the anserine tendon. The diagnostic hypothesis was carpal tunnel syndrome and anserine tendinitis.

The therapeutic approach adopted was a prednisone cascade (40–30–20-10 mg/day). New tests were requested [rheumatoid factor, cyclic citrullinated peptide antibody test (anti-CCP), ESR and CRP]. The patient was referred to physiotherapy, and a new prescription for cyclobenzaprine and gabapentin was ordered.

In January 2023, the patient returned with the requested test results. She reported pain in the cervical and lumbar regions, which radiated to the lower limbs, and pain in the left hip that worsened when walking, going up and down stairs, and when lying down on the left side. She reported morning stiffness of <30-min duration. She was not exercising and was unable to schedule physical therapy. The VNS was 8/10. She mentioned that she was experiencing episodes of sadness and insomnia, sleeping approximately 2–4 h a night. She denied feeling pain in her hands and wrists.

During the physical examination, she reported pain on palpation of the cervical and lumbar paravertebral regions, with negative Lasègue and Spurling tests. She reported pain on palpation of the bilateral hip in the area of the trochanteric bursa. She also reported pain on palpation at the anserine tendon insertion. Lower limb edema (+/4+) was noted. A squeeze test of the feet was positive, while a squeeze test of the hands was negative. There was no arthritis. The diagnostic hypotheses were fibromyalgia, osteoarthritis, anserine tendinitis and trochanteric bursitis.

During the examination, magnetic resonance imaging (MRI) of the cervical spine was requested, the patient was referred for hydrotherapy and physiotherapy, and 25 mg/day amitriptyline was prescribed. In addition, physical exercise was advised, and a referral to a nutritionist was made.

In April 2023, the patient returned with the new test results after using the prescribed medications (cyclobenzaprine 10 mg/day when she had pain and gabapentin 300 mg/day 3 times/day). She reported having completed hydrotherapy and physiotherapy sessions, with partial improvement of symptoms, and she had received guidance from the physiotherapist to participate in a few more sessions. She reported that her lower back pain, shoulder and elbow pain after repetitive effort, and leg pain due to neuropathy were milder than at the previous consultation.

She reported having experienced episodes of sadness, anxiety, insomnia (she did not use amitriptyline due to tremors and a bitter taste in her mouth) and irritability, which were ameliorated after walking. These symptoms also improved with the use of aromatherapy, melatonin, tryptophan and mental exercises with the help of friends and family. She claimed unmeasured weight loss.

She had a consultation with a psychologist and would begin follow-up. She was referred for a scheduled consultation with a psychiatrist, but an anxiety attack prevented her from staying there to carry out the consultation.

On physical examination, there was pain on lumbar paravertebral palpation, and Lasègue’s test was negative. Bilateral pain was reported on palpation of the hip in the area of the trochanteric bursa. Pain was also reported on palpation at the anserine tendon insertion. Lower limb edema (+/4+) was noted. There were no signs/symptoms of arthritis. Pain was reported when moving the shoulders, but without crackles or local redness. The Jobe, Gerber and Patte maneuvers were negative. Pain was reported on palpation of the lateral epicondyle of the left elbow. The Cozen test was positive. The diagnostic hypotheses were fibromyalgia, osteoarthritis, anserine tendinitis, trochanteric bursitis, obesity, lateral epicondylitis and rotator cuff syndrome.

A new referral was made for physiotherapy, hydrotherapy, muscle strengthening and guidance to maintain physical exercise. The patient was instructed to maintain psychological follow-up and to consult with a psychiatrist. Gabapentin and cyclobenzaprine were continued as necessary.

In August 2023, the patient returned and was using 10 mg/day cyclobenzaprine (when she had pain) and 300 mg/day gabapentin twice a day. She reported an improvement in her diffuse muscular pain, but she still had pain in her hips and cervical and lumbar spine, with exertion, but of mild intensity. She underwent physical therapy, which led to significant improvement. She underwent consultation with an orthopedist for her hip pain and underwent MRI of the pelvis (06/15/2023), which identified calcareous peritendinopathy of the gluteus minimus bilaterally, in addition to bilateral insertional tendinopathy of the gluteus minimus. MRI of the lumbar spine revealed T11-T12 disc protrusion without herniation or spinal cord compression. She denied pain in her shoulders and elbows at the time. She reported that she had lost 10 kg of weight since starting treatment.

On examination, there was mild pain on cervical and lumbar paravertebral palpation and a negative Lasègue test. Bilateral pain on palpation of the hip in the area of the trochanteric bursa was reported. Pain was also reported on palpation at the anserine tendon insertion. Lower limb edema (+/4+) was noted. There was no arthritis.

New physiotherapy sessions for the hips and water aerobics were suggested when financially possible. Psychology monitoring was maintained, and consultation with a psychiatrist was recommended. The gabapentin dosage was reduced to 12 mg every 12 h, and cyclobenzaprine could be added as necessary.

### Patient #2

A 33-year-old female student received a serological diagnosis of HTLV-1/2 infection in December 2022 when she donated blood, and HTLV-1 infection was confirmed. In the same year, she was diagnosed with fibromyalgia, and in 2023, she began suffering from arthritis. She attended her first rheumatology appointment in December 2022 with the main complaint of “swelling of the hands and feet.”

Approximately 1 year ago, she reported edema in her hands, feet and face, in addition to diffuse body pain that appeared and lasted a few days, with spontaneous improvement. She reported that these episodes were not related to climate or food. She also reported paresthesia in the upper limbs, unrelated to movement or effort. She complained of pain in her lower limbs and upper limbs at dawn that worsened throughout the day, as well as a feeling of great heaviness in her legs that worsened with physical exertion. She reported nonrestorative sleep. She denied using medications to improve her clinical condition.

She stated that she had an anxiety disorder and was taking 50 mg/day sertraline hydrochloride. She reported walking twice a week for 30 min. There were no previous cases of HTLV infection in the family.

On physical examination, she presented diffuse pain on palpation, without arthritis, but with pain on palpation of all joints of the hands, wrists, elbows, ankles and feet; in the dorsal and lumbar regions; and in the paravertebral region.

Given these findings, the diagnostic hypothesis was fibromyalgia. Laboratory tests (Table 1) were requested (blood count, ESR, C-reactive protein, rheumatoid factor, antinuclear factor, thyroid-stimulating hormone [TSH], and free T4), and psychological, psychiatric and endocrinological follow-up was advised, the latter due to obesity.

In September 2023, the patient returned for consultation, but without the results of the requested tests. At this time, she was using sertraline hydrochloride, had persistent anxiety, and reported having generalized muscle pain for 2 weeks, which caused difficulty sleeping and repeated awakening. Approximately 2 months before, she started experiencing joint pain in her hands, which worsened upon exertion, and morning stiffness (<30 min), in addition to pain in the cervical and lumbar regions, with difficulty in squatting. She reported photosensitivity resulting in melasma.

On physical examination, there was diffuse pain on palpation and arthritis in the left wrist and right fourth proximal interphalangeal joint (4th PIP). There was pain in all joints of the hands, wrists, elbows, ankles and feet. A squeeze test of the hands was positive. Pain was reported on palpation of the dorsal and lumbar region in the paravertebral region. The patient exhibited pain upon palpation and mobilization of the cervical region and a negative Spurling test. The Clinical Disease Activity Index (CDAI) was 40.

After this consultation, in addition to fibromyalgia, rheumatoid arthritis (RA) and systemic lupus erythematosus (SLE) were considered. ANA, rheumatoid factor, ESR, CRP, blood count, TSH and free T4 tests were requested, as were X-rays of the hands and lumbar spine. Pregabalin (75 mg every 12 h) was prescribed.

In October 2023, the patient returned for consultation, reporting a slight improvement in her pain with the use of pregabalin. She still did not have the test results. The joint pain persisted with worsening morning stiffness (> 30 min), and she reported difficulty cutting food due to loss of strength in her left hand. She also reported having difficulty sleeping.

Physical examination revealed diffuse pain on palpation; arthritis in the left wrist and right 4th PIP; and pain in all joints of the hands, wrists and ankles. A squeeze test of the hands was positive. Arthritis was not present in the knees. Lower limb edema (++/4+) was noted. There were 10+ tender points, and the CDAI was 36.

A prednisone cascade of 40–30–20-10 mg and methotrexate 15 mg/week combined with folic acid 5 mg/week were prescribed. Serologies for hepatitis B and C and HIV-1 and -2 were requested, in addition to general laboratory tests.

The patient returned for consultation in March 2024 with polyarthritis, maintaining the suspected diagnosis of rheumatoid arthritis, and taking 15 mg methotrexate. She complained of pain in her hands and arms, with fatigue for approximately 2 weeks. There was persistent insomnia and episodes of morning stiffness with difficulty carrying objects.

On physical examination, the patient had arthritis in the wrists, 4th and 5th PIP, positive squeeze tests of the hands and feet, and pain on palpation of the upper and lower limbs. The CDAI of 29 was indicative of high disease activity.

During management, the dose of methotrexate was increased to 20 mg per week, a new cascade of prednisone was prescribed, pregabalin was reintroduced, and zolpidem (10 mg per day) was prescribed. The patient was referred to a psychologist. The patient is currently awaiting the results of tests to confirm the diagnosis of polyarthritis.

### Patient #3

A 50-year-old female was serologically diagnosed with HTLV-1/2 infection in 2016 when she underwent routine tests during blood donation. In 2017, she was diagnosed with fibromyalgia, and in 2022, she developed osteoarthritis in her right knee. In 2023, HTLV-2 infection was confirmed.

In November 2022, she attended her first rheumatology consultation, where she reported experiencing diffuse body pain daily for approximately 6 years, with greater intensity at the end of the day [numeric verbal scale (NVS) = 8]; morning stiffness <30 min; restful sleep with the use of pregabalin (100 mg/day); and irregular use of duloxetine hydrochloride. Despite experiencing periods of anxiety, her daily activities were not affected. When asked, she denied cases of HTLV infection in her family, as well as the use of illicit drugs, smoking and alcohol consumption. The patient was not sedentary and practiced water aerobics twice a week. On physical examination, no synovitis was observed in the hands. She presented diffuse pain on generalized muscle palpation, and crepitus in both knees but no arthritis. The Zohlen test was positive in the right knee. The diagnostic hypotheses were fibromyalgia and chondromalacia. At the end of the consultation, laboratory tests (Table 1) (blood count, ESR, C-reactive protein, rheumatoid factor, and antinuclear factor) and knee MRI were requested.

In April 2023, the patient returned with the requested test results. She reported that the pain in her right knee improved significantly after starting water aerobics, appearing only when she exerted a great deal of effort on the joint during physical exercise. She reported that her sleep was restful, and her body pain improved slightly after 45 days of increasing the dose of pregabalin to 150 mg/day (NVS = 7). The patient reported that for approximately 2 weeks, she had been experiencing pain in the lower back at the end of the day, which worsened with exertion and when carrying weight, alternating between the right and left sides. She reported that the pain sometimes radiated to her lower limbs and denied paresthesia. She also reported episodes of paresthesia in her upper limbs, which appeared at night while she was sleeping.

On physical examination, she presented pain with shoulder movement, especially on the right, with worsening during external and internal rotations. The Gerber and Patte tests were positive. Pain was noted upon mobilization of the lumbar spine, especially upon hyperextension, with pain upon palpation of the right lumbar paravertebral region. The Lasègue sign was negative. The patient reported pain on palpation of the greater trochanter region of the femur, bilaterally, in the area of the trochanteric bursa.

The therapeutic approach was to increase the frequency of water aerobics to 3 times a week and use 10 mg/day cyclobenzaprine. The possibility of increasing the pregabalin dose to 300 mg/day was considered, but the patient preferred something less expensive; therefore, the doctor opted for cyclobenzaprine.

### Patient #4

A 53-year-old female with type 2 diabetes was treated with 500 mg metformin hydrochloride and had a previous serological screening for HTLV-1/2 in 2021. In 2022, she was diagnosed with fibromyalgia, and in 2023, the infection by HTLV-2 was confirmed.

In April 2022, she attended her first consultation with a rheumatologist, presenting diffuse pain throughout her body. For approximately 5 years, the patient had complained of pain in her knees, legs, arms, and hands, as well as episodes of weakness and malaise with greater intensity at the end of the day. She had prolonged morning stiffness. The pain improved when she rested and worsened with exertion (VNS = 10). The patient also reported pain and paresthesia in the hands, especially in the morning, upon waking. She reported nonrestorative sleep, awakening approximately three time per night. She reported symptoms of sadness (cried easily). She mentioned difficulty carrying out activities due to pain and discomfort in her legs, with edema at the end of the day and episodes of paresthesia. Furthermore, she denied cases of HTLV-1/2 in her family, as well as the use of legal or illicit drugs. She reported being sedentary.

On physical examination, there was pain on palpation of the hand joints, but no synovitis was observed. A squeeze test of the hands was positive. There was pain on palpation of the metatarsophalangeal (MTP) joint, and a squeeze test was positive bilaterally. Lower limb edema (+/4+) was noted. Shoulder pain was noted on mobilization, with reduced mobility in the right shoulder (motorcycle accident in January 2022), and diffuse muscle pain was noted on palpation. Laboratory tests (Table 1) (blood count, ESR, C-reactive protein, blood glucose, total cholesterol, LDL, HDL, triglycerides, rheumatoid factor, and glycated hemoglobin) and X-rays of the hands and feet were requested.

The return consultation, with presentation of the results of the requested tests, took place in November 2022. The patient continued to experience diffuse pain, especially in the right hip and cervical spine. Furthermore, she consulted a rheumatologist in September. She received a diagnosis of rheumatoid arthritis and was prescribed methotrexate 10 mg/week, but the patient was confused about the dose and used only 1 tablet/week instead of 4 tablets/week. The patient complained of nonrestorative sleep, pain in the hands, morning stiffness <30 min and VNS = 10. She reported persistent anxiety symptoms, with increased hunger and sadness. On physical examination, she presented diffuse pain upon palpation. There was no arthritis or enthesitis in the hands or feet or pain on palpation of the lumbar paravertebral region. Lasègue’s sign was negative. There was pain on bilateral hip mobilization, and Patrick’s test for the coxofemoral joint was positive.

The diagnostic hypothesis was fibromyalgia, and the following drugs were prescribed: sertraline hydrochloride 50 mg/day, pregabalin 75 mg/day and a suspension of methotrexate (MTX) and folic acid for rheumatoid arthritis. Furthermore, new tests were requested (blood count, ESR, C-reactive protein, urea, creatinine, blood glucose, glycated hemoglobin, total cholesterol and fractions and triglycerides), as were radiography of the lumbar spine and pelvis.

In September 2023, the patient returned with diffuse pain, but this pain, mainly in the dorsal and lumbar regions, worsened with exertion and improved with rest. At night, when sleeping, she experienced throbbing pain in her lower back and was unable to find a position to sleep, staying awake most of the night. She still had paresthesia in her hands and feet.

On physical examination, she presented diffuse pain on palpation, with no arthritis or enthesitis in her hands or feet. There was pain in the cervical spine on palpation and local mobilization, pain in the dorsal spine on paravertebral palpation, and pain in the lumbar spine on palpation of the paravertebral region. Pain with movement was noted for extension, flexion and lateralization. The Tinel and Phalen tests were negative, with evidence of diffuse edema on the dorsum of the left foot. The Cacifo (Godet) sign was positive in the lower limbs (+/4+). At the time of the consultation, the patient was taking the following medications: metformin 850 mg 3 times/day, simvastatin 20 mg/day, and insulin (Hagedorn’s neutral protamine - HNP) 24 IU in the morning and 12 IU at night. The use of sertraline hydrochloride 50 mg/day and pregabalin 75 mg/day was maintained. Radiographs of the lumbar, cervical and dorsal spines were requested, in addition to new laboratory tests (blood count, ESR, C-reactive protein, ANA, rheumatoid factor, TSH and free T4). The patient was referred to physiotherapy upon return and advised to perform low-impact aerobic physical exercise, and follow-up with a psychologist was recommended.

In October 2023, at the return visit, she reported that there was only partial improvement in body pain after using sertraline hydrochloride. Her insomnia, complaint of inability to find a comfortable position to sleep and tingling in the hands and feet persisted. She reported that it was not possible to consult a psychologist as advised.

At this time, the patient was taking the following medications: metformin 850 mg 3 times/day, simvastatin 20 mg/day, HNP insulin 24 IU in the morning and 12 IU at night, and sertraline hydrochloride 50 mg/day.

On physical examination, she had diffuse palpation pain. There were no signs of arthritis or enthesitis in the hands or feet, but there was diffuse edema on the dorsum of the left foot. The Lock sign was positive in the lower limbs (+/4+).

After clinical evaluation and examinations, two other diagnostic hypotheses were considered in addition to fibromyalgia: decompensated diabetes or diabetic neuropathy. Sertraline hydrochloride was maintained, and 25 mg/day amitriptyline was introduced. The patient was advised to return as soon as possible to optimize the treatment of diabetes mellitus.

## Discussion

Fibromyalgia is a complex disease for which the etiology remains unknown. In the present study, we describe four patients with fibromyalgia associated with HTLV-1 and HTLV-2 infection. Two patients were women over 50 years of age with confirmed HTLV-2 infection who presented signs and symptoms of fibromyalgia and other rheumatological manifestations. Contrary to what has already been described for HTLV-1 (4), the association of HTLV-2 with diseases is still rare, and further clinical studies are needed, especially in regions where HTLV-2 is endemic. There are currently few reports of an association between infection and neurological disease (5, 16) and only one report of a high prevalence of HTLV-1 infection in a cohort of patients with fibromyalgia (13), with no reports associating the disease with HTLV-2 infection.

HTLV-1 and HTLV-2 infections are chronic, silent (17), and considered asymptomatic in the majority of diagnosed cases (18). HTLV-1 infection has been reported to cause morbidity and mortality in 5–10% of infected people (19). These characteristics have led this retrovirus to be neglected (20, 21), especially in regards to therapeutic and immunoprevention strategies (22, 23). HTLV-2, in turn, is endemic in vulnerable populations that are difficult to access (24, 25), and as it is considered an older virus adapted to the human host, HTLV-2 has been neglected and little studied for its possible role in the development of inflammatory diseases. In this sense, our group has been working on clinical monitoring of confirmed cases of HTLV-1/2 infection and actively searching for infection by these viral types in patients diagnosed with inflammatory diseases, which resulted in the description of these patients whose cases are first reported here.

Another problem associated with this infection is related to the stigma of a sexually transmitted infection (STI) (26), an aspect still little explored in the assessment of people living with HTLV-1/2. Some patients choose to omit the diagnosis of HTLV and request confidentiality about the infection, as there is still a feeling of inferiority and judgment (15). The rejection that infected patients may feel is also related to work difficulties, as the current job market becomes more competitive every day. If the desired activity is not carried out, it is natural for feelings of incapacity and inferiority to arise (27, 28). Thus, a lack of independence, consequently, generates feelings of sadness and anguish that lead to depression (28–30). In two of the cases reported here, the patients used antidepressant medications such as sertraline hydrochloride and duloxetine hydrochloride in addition to pregabalin.

In addition to the possibility of depression, the impact generated by learning about infection with a little-known virus can cause posttraumatic stress disorder (PTSD), characterized by a set of behavioral and emotional signs and symptoms of intense fear, terror or hopelessness (31). These psychological symptoms tend to increase somatic and physical complaints, and consequently, the incidence of fibromyalgia in these cases of PTSD is high. Some studies indicate that there is a relationship between fibromyalgia and the psychosomatic symptoms of PTSD (32, 33). Furthermore, there is evidence highlighting a link between PTSD and inflammatory changes, with significantly increased rates of physical comorbidities in which immune dysregulation is involved (34). Given the complexity of this process, there is evidence that viral infections could trigger the dysregulation of the inflammatory response and the impairment of the nervous system in patients with fibromyalgia (35, 36). In this context, HTLV-1 infection has also been associated with cognitive changes under the influence of an inflammatory immune response (37). Unfortunately, it was not possible to measure the serum levels of proinflammatory cytokines as well as a follow-up of the proviral load in the patients described here, in order to determine the inflammatory status and viral replication level, which can be considered a limitation of our study.

The open question is whether HTLV-2 could also induce the changes already observed for HTLV-1. Our results show that 23% of individuals infected with both viral types presented rheumatological findings with complaints of diffuse pain. This percentage is >5–10% reported in the literature for cases of symptomatic HTLV-1 infection, which leads us to infer that the frequency of symptoms associated with infection by these viruses is greater and necessitates a thorough clinical evaluation of all those infected. Furthermore, if we consider only cases of HTLV-2 infection, which has historically been described as asymptomatic, four of our patients had rheumatological complaints and of two of these patients had a diagnosis of fibromyalgia. These results have led us to rethink the established idea that HTLV-2 is not associated with disease.

Fibromyalgia is a chronic medical condition that affects millions of people around the world, leading to a search for a better understanding of the causes and the possible association with viral infections. In this sense, the results presented here contribute by providing a new perspective to rheumatologists in the interpretation, understanding, elucidation of the diagnosis and treatment of their patients’ pain syndromes, while at the same time providing a greater response to the complaints expressed by society. Another aspect of the benefit of the present study is the possibility of reducing costs for the public health service, since the rheumatologist, when identifying the infection in a person with a rheumatological condition, can minimize the costs of searching for imaging tests, in addition to being able to direct symptomatic patients to treat pain, whether through medication or physical activities, such as physiotherapy and Pilates. Thus, our findings have practical relevance, as they provide a different perspective for rheumatologists when seeking to diagnose HTLV infection in patients with fibromyalgia. Furthermore, the results highlight the importance of including HTLV-1/2 in medical education programs, as well as for patients with fibromyalgia, such as the Health Education program for fibromyalgia, like the one already existing in Brazil called “Fibro Friends” (38).

## Conclusion

Considering the clinical cases presented here and, above all, the first reports of patients with HTLV-2 infection with a clinical picture of fibromyalgia, the importance of in-depth studies of the pathogenicity of this viral type, similar to what have already been carried out for HTLV-1, is highlighted. Carrying out cohort studies with more detailed clinical monitoring of cases of HTLV-2 infection is necessary, as evidence today suggests that HTLV-1 is the cause of disease in more than 5% of those infected, contradicting the initial estimates. The idea that HTLV-2 infection is benign may not be entirely true for some individuals, especially in those living in populations in which HTLV-2 infection is hyperendemic and public health services are deficient, as is the case in indigenous populations of the Brazilian Amazon (39).

## Data Availability

The raw data supporting the conclusions of this article will be made available by the authors, without undue reservation.
